# Bevacizumab Combined With Oxaliplatin/Capecitabine in Patient With Refractory and Recurrent Mucinous Adenocarcinoma of the Appendix: A Case Report

**DOI:** 10.3389/fonc.2019.00055

**Published:** 2019-02-07

**Authors:** Wenzhi Liu, Lili Liu, Ruoyu Wang, Guanyu Gong, Xinjia Ding, Bin Yang, Yun Bao, Zhiqiang Wang, Bo Zhang, Dewei Zhao, Fei Wu, Yan Ding

**Affiliations:** ^1^The Institute for Translational Medicine, The Affiliated Zhongshan Hospital of Dalian University, Dalian, China; ^2^Department of Oncology, The Second Affiliated Hospital of Dalian Medical University, Dalian, China; ^3^Genomic Future Inc., Lexington, MA, United States; ^4^Department of Neurosurgery, The Second Affiliated Hospital of Dalian Medical University, Dalian, China; ^5^Department of Pediatrics, Children's Hospital of Boston, Harvard Medical School, Boston, MA, United States

**Keywords:** appendiceal adenocarcinoma, peritoneal carcinomatosis, next generation sequencing, bevacizumab, targeted therapy

## Abstract

Primary appendiceal adenocarcinoma with peritoneal pseudomyxoma (PPM) has a high recurrence rate and refractory to medical interventions such as repetitive debulking surgery and systemic chemotherapy. Genome-based targeted therapy for such cases has not been well-documented. Here we present a 63-years-old women, who was diagnosed with recurrent mucinous adenocarcinoma of the appendix with local invasions and peritoneal carcinomatosis, was refractory to systemic chemotherapy after surgery. We used a regime developed using whole exome sequencing. Somatic mutations in the genes encoding VEGFR2, FGFR1, FGFR2, FGFR3, and KRAS were identified in the patient's tumor tissue. The patient was then treated with bevacizumab plus oxaliplatin. After 4 months of treatment, pelvic CT showed dramatic reduction of pseudomyoma and a decline of CA199 level from 5436.7 to 1121.4 U/ml. Continual treatment with bevacizumab-capecitabine remained effective and the patient's CA199 level further decreased to 401.26 U/ml according to the follow-up examination on Aug 15th, 2018. Results from this study show the evidence of gene mutations involving VEGF signal activation in the recurrence of appendiceal adenocarcinoma. Our results also suggest the association of these mutations with the effectiveness of anti-VEGF treatment using bevacizumab. Therefore, the screening of gene mutations involved in VEGF signaling and targeted therapy with anti-VEGF drugs may provide a new option to manage refractory/recurrent advanced-stage appendiceal adenocarcinoma.

## Background

Primary adenocarcinoma of the appendix is a rare malignancy and accounts for 0.4% of gastrointestinal tumors, according to a report of national cancer institute (NCI) (1). Mucinous adenocarcinoma is the most common histological subtype (37%), followed by colonic and carcinoid subtypes (2). The clinical presentations of appendiceal cancer are vague until advanced stage. As a result, early diagnosis of appendiceal cancer is often difficult. Common complications of late stage disease include rupture and acute appendicitis (accounting for ~1% appendectomy cases) (3), local invasion and peritoneal carcinomatosis (PC)/peritoneal pseudomyxoma (PPM) (4, 5). The advanced stage has a poor overall survival rate with median survival time of 5.2–12.6 months (5). Currently there is no standard medical care for the disseminated late-stage appendiceal cancer with PC/PPM. It has been generally recommended to perform cytoreductive surgery (CRS) combined with perioperative hyperthermic intraperitoneal chemotherapy (HIPEC) or postoperative intraperitoneal chemotherapy (EPIC) with mitomycin C, cisplatin, 5-FU, or a combination (5, 6). Unfortunately, most appendiceal cancer patients with PC/PPM experience recurrent and refractory to treatment, and fail to repetitive surgery and systemic chemotherapy (6).

Targeted therapy has been successfully used to treat many types of cancers including colorectal cancer. However, to the best of our knowledge, genome-based targeted therapy for the appendiceal cancer has never been reported. In the present case, a patient was diagnosed with mucinous adenocarcinoma of the appendix with peritoneal carcinomatosis and multiple local invasions. The patient received routine treatments by CRS-HEPIC-EPIC but relapsed after 1 year. Then the patient's condition deteriorated continuously and experienced recurrent and refractory to the treatment. Using whole exome sequencing and targeted medicine, optimal therapeutical efficacy was achieved with a gradual remission and remains progression-free until now.

## Case Presentation

**Figure 1:**
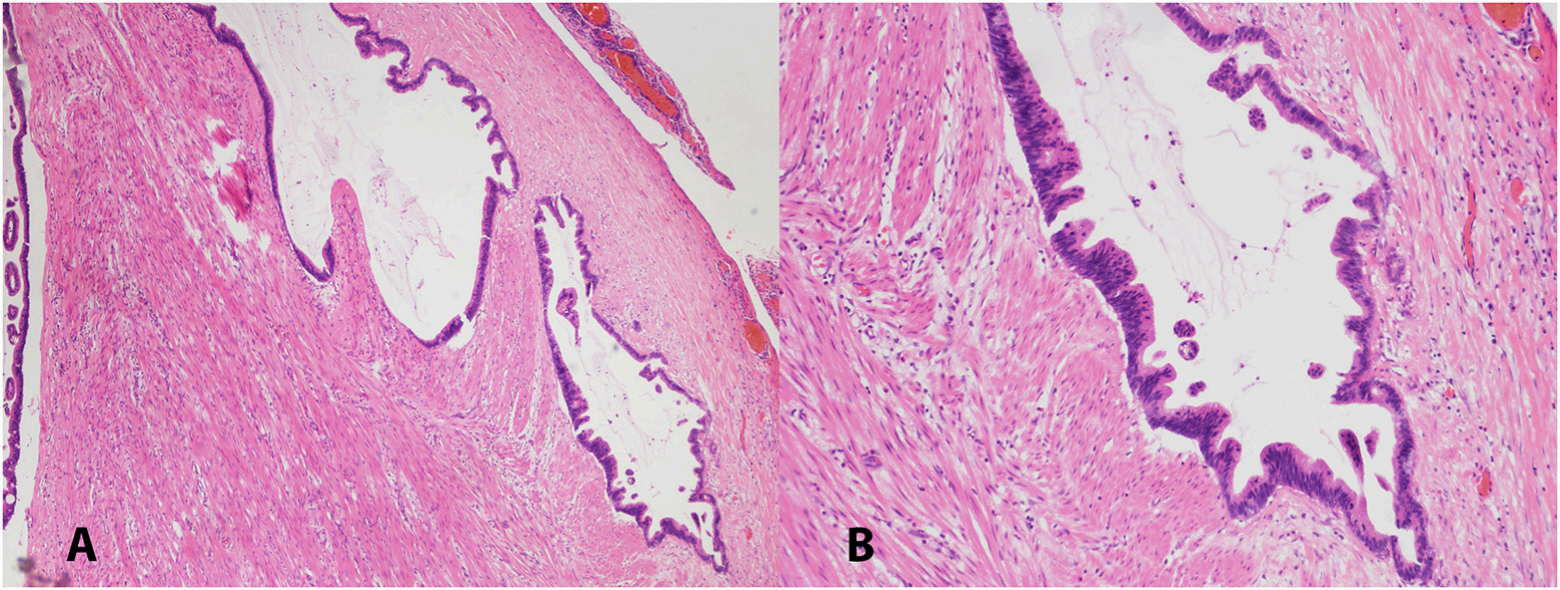
Low (**A**, 40X) and high (**B**, 100X) magnification pictures of appendiceal mucinous adenocarcinoma. H&E stained.

**Figure 2:**
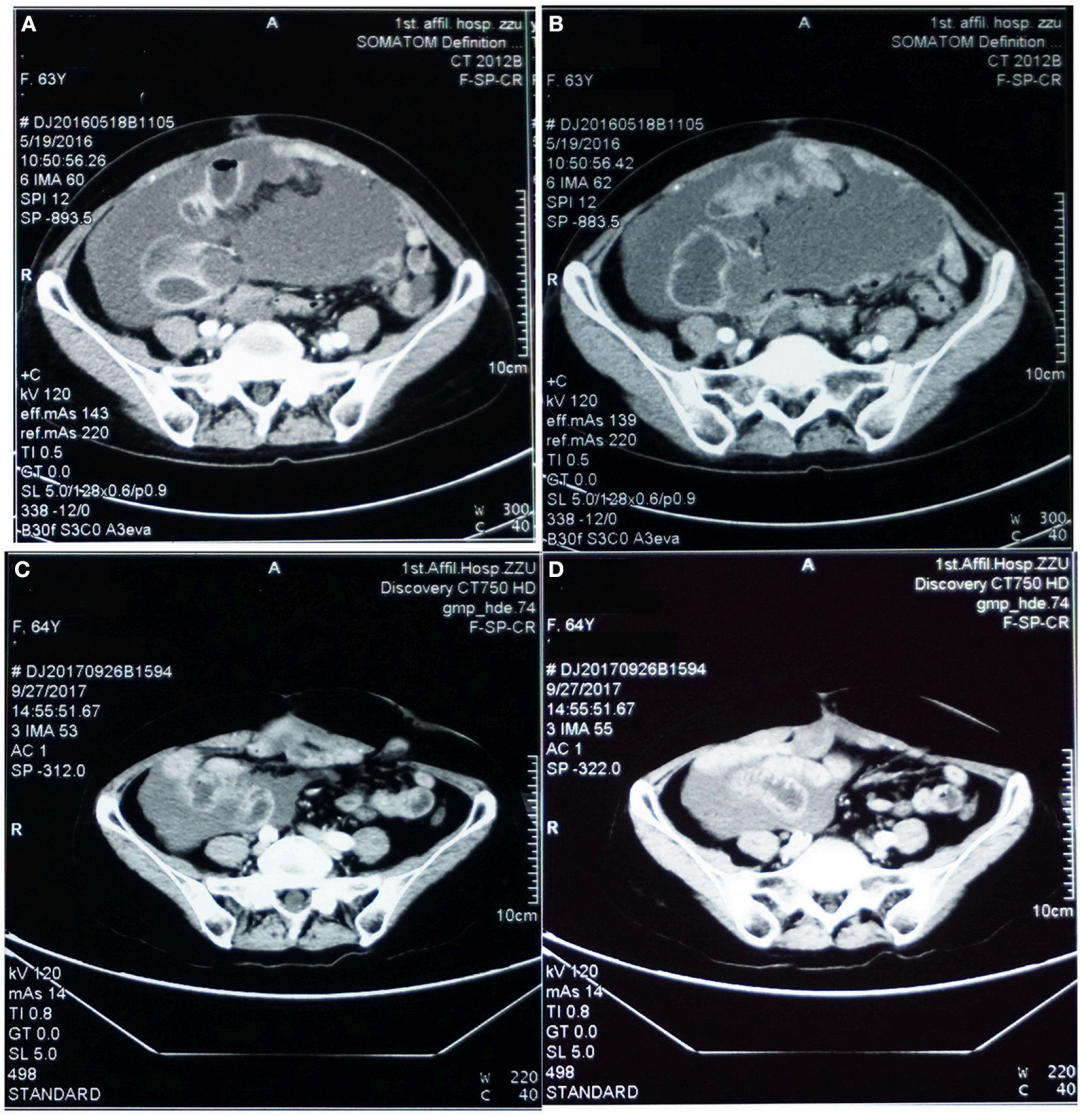
Pelvic CT images before **(A,B)** vs. after **(C,D)** targeted therapy. **(A,B)** Prior to targeted therapy, images showed intraperitoneal multiple nodules, and a large number of peritoneal cavities filled up with mucus. **(C,D)** After targeted therapy using bevacizumab and oxaliplatin, image on the same sections showed significantly reduced peritoneal nodules and mucous cavities, suggesting stabilization of disease progression and partial response.

A 63-years-old Chinese female presented with asymptomatic palpable abdominal mass, increased carbohydrate antigen 19-9 (CA-199) level and pelvic mass on CT scan. An opening surgery observed an appendiceal mass involving the entire layer of the appendix, rupture, invasion of bilateral ovaries, wide-spreading nodular implantations with pseudomyxoma in peritoneal cavity, greater omentum, small intestine mesentery and hepatic and splenic regions. Debulking surgery with peritoneal nodule ablation and mucus reduction was performed in Beijing 301 Hospital. Postoperative pathology confirmed mucinous adenocarcinoma of the appendix T4NxM1, stage IV with peritoneal carcinomatosis (Figure 1). After surgery, the patient received one time standard perioperative hyperthermic intraperitoneal chemotherapy (HIPEC) with mitomycin C. Because of the excessive peritoneal carcinomatosis, the patient was given three cycles of postoperative intraperitoneal chemotherapy (EPIC) with 5-FU plus mitomycin C. The patient remained symptom free for 1 year until she developed progressive abdominal distension, loss of appetite and worsening nourishment. The patient failed to response to further systemic chemotherapy, and a large number of PPM (Figures 2A,B). Then a second surgery was performed, intestinal obstruction by mucous cavities was observed, and a colostomy was given. Shortly after operation, cetuximab, a monoclonal antibody binding to and inhibiting EGFR, was given to the patient for 20 days (yet without gene testing) at a local hospital, but failed to show any improvement. By then the patient had tried all available approved options and became refractory to the treatments.

At the time when the patient visited us, she was severely wasted, with progressive abdominal distension and elevated CA-199 level at 5436.7 U/ml. Considering her weak constitution and failure of previous interventions, alternative treatment strategies, especially a rationally designed targeted therapy, emerged to be the last-ditch option to the patient. Targeted therapy is usually based on a patient's genomic profile by genetic testing. In order to find the accurate target, we decided to use the paraffin-embedded surgical tumor tissue from the patient, and detect gene mutations using the TruSeq Rapid Capture Exome Kit for whole exome sequencing (WES) on the Illumina NextSeq500 sequencing platform. The WES data was then analyzed using OncoDecoder™ (Genomic Future, Inc. USA). Several key gene mutations were identified including a missense mutation p.Gln472His (exon 11) in KDR/VEGFR-2, a missense mutation p.Arg281Gln (exon 8) in FGFR1, a missense mutation p.Lys296Arg (exon 7) in FGFR2, a missense mutation p.Thr654Ser (exon 14) in FGFR3 and a missense mutation p.Gly12Asp (exon 2) in KRAS. Additional 38 gene mutations including TP53, ERBB2, KIT, GNA11, and JAK3 were also detected (Table 1).

**Table 1:** Mutated genes identified in the present case of mucinous adenocarcinoma.

**AKT1**	**ATM**	**CSFIR**	**ERBB2**	**FGFR1**	**FGFR2**	**FGFR3**	**FLT3**
**GNA11**	**JAK3**	**KDR**	**KIT**	**KRAS**	**NOTCH1**	**PIK3CA**	**PET**
**SMARCB1**	**SMO**	**STK11**	**TP53**				

Although no NCCN-guided targeted therapy regime for appendiceal mucinous adenocarcinoma is documented as of to-date, there are two approved drugs for colorectal cancer may be considered as potential candidates: bevacizumab and cetuximab. Bevacizumab is a monoclonal antibody blocking the VEGF ligand, and bevacizumab in combination with standard chemotherapy has been approved by FDA as first line treatment for metastatic colorectal cancer (7, 8). We predicted that bevacizumab may be a suitable targeted drug candidate for our case based on the following three reasons: Firstly, the gene testing results showed several mutations involving KDR/VEGFR-2, FGFR1, FGFR2, and FGFR3. Although these mutations are currently classified as variation of uncertain significance (VUS), hyperactive VEGF pathway is a common event in colorectal cancer and contributes to tumor metastatic activity (9). A recent study from the MD Anderson cancer center showed improved average overall survival and progression-free survival by providing anti-VEGF treatment to patients diagnosed with unresectable appendiceal epithelial neoplasm (yet no gene testing was performed) (10). This finding suggests that VEGF hyperactivity could be a common event in appendiceal cancer, and bevacizumab could be a promising targeted drug. Next, it has been known that the efficacy of certain EGFR monoclonal antibody drugs, including cetuximab and panitumumab, could be affected by KRAS mutation (6). Indeed, in the present case, we identified KRAS p.Gly12Asp missense mutation, which could cause inefficient response to cetuximab (11). However, the efficacy of bevacizumab for colorectal cancer treatment has been testified to be independent from KRAS mutation (8). Thirdly, there was no contraindication of bevacizumab usage to the patient. The common risk factors include low WBC count, high blood pressure, impaired heart function and poor renal function.

Under our advice, the patient received treatment of bevacizumab (7.5 mg/Kg, in total 450 mg, IV-GTT) plus oxaliplatin (130 mg/m^2^, in total 200 mg IV-GTT) on day 1 every 3 weeks for 6 cycles since August, 2016. Follow-up examination after treatment showed significant improvement of the patient's condition, and CT scan imaging results showed dramatic reduction of her peritoneal mucus (as shown in Figures 2C,D). In addition, the patient's CA-199 level decreased from 5,436.7 U/ml (before treatment) to 1121.4 U/ml (after treatment). Afterwards, the patient received continuous maintenance treatment using bevacizumab (7.5 mg/Kg, in total 450 mg, IV-GTT on day 1 each 3 weeks) plus capecitabine (1,500 mg, oral, twice a day) for days 1 to 14 until now. The patient has been followed up routinely to evaluate the treatment efficacy and to monitor the adverse effects. The main adverse effects were numbness in the hands and feet, dry nose and epistaxis, dry throat, fatigue, loss of appetite. The patient has been progression-free as of recent follow-up on September 26th, 2018 with the most recent CA-199 being 401.26 U/ml on August 15th, 2018.

## Discussion

Primary adenocarcinoma of the appendix is a rare neoplasm with an incidence of 1.2 cases per 100,000 people each year (12). The prognosis of appendiceal adenocarcinoma varies depending on the histology types, including colonic-type adenocarcinoma, typical carcinoid, mucinous adenocarcinoma, and singlet ring cell adenocarcinoma (3). The mucinous adenocarcinoma is similar to the ovarian adenocarcinoma, and peritoneal dissemination is a frequent metastatic route (12). Like most colorectal cancers, the appendiceal adenocarcinoma presents with non-specific symptoms and is difficult to be diagnosed preoperatively (4, 5). As a result, it is often found at an advanced stage in which the disease has already spread within abdomen (5). Appendiceal adenocarcinoma-derived peritoneal carcinomatosis (PC) or peritoneal pseudomyxoma (PPM) is a very poor prognostic factor with average life expectancy between half and 1 year (5). In the present case, the patient presented with asymptomatic abdominal mass, local invasions to greater omentum and fallopian tubes and peritoneal carcinomatosis with multiple pseudomyxoma cavities at the initial visit.

The management of mucinous appendiceal adenocarcinoma varies depending on the stages and does not have standard guideline. Right hemicolectomy remains the treatment of choice for the early stage local or regional appendiceal adenocarcinoma (5, 6). CRS-HIPEC or EPIC is usually recommended for the appendiceal or colonic-type carcinoma with confined peritoneal metastasis (5). However, there is no standardized protocol for HIPEC or EPIC (5), and it only achieved complete response in some patients (5, 6). In our case, the patient received CRS-HIPEC-EPIC regime but relapse of peritoneal carcinomatosis occurred 1 year later. Systemic 5-fluorouracil-based chemotherapy was barely beneficial.

Recently, genome-based precision medicine has made great progress to treat a variety of cancers, including colorectal cancer (13). Targeted drugs feature high efficiency and low toxicity. To the best of our knowledge, genome-based targeted therapy for metastatic appendiceal adenocarcinoma has not yet been reported. In order to seek for appropriate targeted therapy for the patient with recurrent and refractory appendiceal cancer, we performed whole exome sequencing with the patient surgical pathology tissue at our genetic testing lab. Several candidate target gene mutations involved in the angiogenesis pathway including KDR/VEGFR-2 and FGFR1, FGFR2, FGFR3 were identified. Both VEGF and FGF pathways function as angiogenetic mediators to promote metastasis of many neoplasms (9). Based on the gene mutation profile, the patient received the bevacizumab-oxaliplatin regime and then the bevacizumab-capecitabine as maintenance treatment. The results showed great effectiveness of the treatment and the patient remains progression-free and continuous decrease of CA-199 level as of to-date. The use of bevacizumab for metastatic appendiceal cancer treatment has been reported in a recent study (10). However, the treatment achieved therapeutical benefits in some patients but not the others, owing to the fact that no gene testing was performed before treatment (10). Therefore, our case report is the first study demonstrating evidence-based therapy for metastatic mucinous appendiceal adenocarcinoma. Indeed, we argue that certain level of cost-effective gene testing may be necessary prior to administration of targeted drugs in order to avoid the abuse of targeted medicines. A good example could be found in our case that the blind usage of anti-EGFR drug cetuximab without prior detection of KRAS mutation from the patient pathology tissue failed to achieve any treatment benefit.

In conclusion, accurate detection of gene mutation can help clinicians to make the optimal choice of individualized targeted drugs, and improve the prognosis and life quality of patients. The present report is one case and limited and waits for more cases to be filled in to expand our knowledge about the genome mutations and personalized medicine of appendiceal cancer.

## Ethics Statement

The patient of this case report agreed and provided written informed consent in accordance with the Declaration of Helsinki. Written informed consent was obtained from the patient for the purpose of publication of the present case report and any relevant images.

## Author Contributions

All authors listed have made a substantial, direct and intellectual contribution to the work, and approved it for publication.

### Conflict of Interest Statement

The authors declare that the research was conducted in the absence of any commercial or financial relationships that could be construed as a potential conflict of interest.

## Abbreviations

CT: computed tomography
PC: peritoneal carcinomatosis
PPM: peritoneal pseudomyxoma
CRS: cytoreductive surgery
HIPEC: hyperthermic intraperitoneal chemotherapy
EPIC: intraperitoneal chemotherapy
VEGFR: Vascular endothelial growth factor receptor
FGFR: Fibroblast growth factor receptor.
